# Family History and Risk of Upper Gastrointestinal Cancer in the Linxian General Population

**DOI:** 10.3389/fonc.2021.605106

**Published:** 2021-05-28

**Authors:** Huan Yang, Jian-bing Wang, Jin-yu Zhang, Jin-hu Fan, You-lin Qiao, Philip R. Taylor

**Affiliations:** ^1^ Department of Cancer Epidemiology, National Cancer Center/National Clinical Research Center for Cancer/Cancer Hospital, Chinese Academy of Medical Sciences and Peking Union Medical College, Beijing, China; ^2^ Department of Epidemiology and Biostatistics, The Children’s Hospital, National Clinical Research Center for Child Health, Zhejiang University School of Medicine, Hangzhou, China; ^3^ Metabolic Epidemiology Branch, Division of Cancer Epidemiology & Genetics, National Cancer Institute, National Institutes of Health, Bethesda, MD, United States

**Keywords:** family history, risk factors, upper gastrointestinal cancer, cohort study, Linxian

## Abstract

**Objective:**

The objective of this study was to investigate family history (FH) of upper gastrointestinal (UGI) cancer and risk of esophageal squamous cell carcinoma (ESCC), gastric cardia carcinoma (GCC), and gastric non-cardia carcinoma (GNCC) in the Linxian General Population Nutrition Intervention Trial (NIT) cohort. Methods: This prospective analysis was conducted using the Linxian NIT cohort data. Subjects with FH of UGI cancer was treated as an exposed group while the remainders were considered as a comparison group. Hazard ratios (HRs) and 95% confidence intervals (CIs) for the association between FH of UGI cancer and risk of UGI cancer incidence and mortality were estimated using Cox proportional hazards models.

**Results:**

There were 5,680 newly diagnosed UGI cancer cases during the follow-up period, with a total of 4,573 UGI cancer deaths occurred, including 2,603 ESCC, 1,410 GCC, and 560 GNCC deaths. A positive FH of UGI cancer was associated with a significantly increased risk of ESCC and GCC (Incidence: HR_ESCC_ = 1.45, 95%CI: 1.35–1.56; HR_GCC_ = 1.27, 95%CI: 1.15–1.40; Mortality: HR_ESCC_ = 1.40, 95%CI: 1.30–1.52; HR_GCC_ = 1.27, 95%CI: 1.14–1.42) after adjusting for age at baseline, gender, smoking status, alcohol drinking, education level, and frequency of fresh fruit and vegetable consumption. Subjects with FH in both parents had the highest risk of ESCC and GCC incidence (HR_ESCC_ = 1.65, 95%CI: 1.40–1.95; HR_GCC_ = 1.42, 95%CI: 1.12–1.81) and deaths (HR_ESCC_ = 1.65, 95%CI: 1.38–1.97; HR_GCC_ = 1.42, 95%CI: 1.09–1.85). Spouse diagnosed with UGI cancer did not increase the risk of any UGI cancers of the subjects. In subgroup analysis, FH of UGI cancer was shown to significantly increase the risk of GCC in non-drinkers (Incidence: HR = 1.31, 95%CI: 1.17–1.47; Mortality: HR = 1.33, 95%CI: 1.17–1.50). No associations were observed for risk of GNCC. Sensitivity analysis by excluding subjects who were followed up less than three years did not materially alter our results.

**Conclusion:**

Our data point to the role of the FH of UGI cancer to the risk of ESCC and GCC incidence and mortality. The influence of family history on the risk of UGI cancer varies from different types of family members.

## Introduction

Upper gastrointestinal (UGI) cancer, including gastric and esophageal cancers, is among the top five leading causes of cancer mortality in China (1). However, considerable differences of UGI cancer incidence exist based on geographic and racial differences, which can be linked to differences in risk factors (2, 3). In Western countries, incidence rates of adenocarcinomas of the esophagus (EA) and gastric cardia (GCA) have been rising rapidly, which may be related to obesity, smoking, and gastroesophageal reflux disease (4). Compared with adenocarcinoma, esophageal squamous cell carcinoma (ESCC) and gastric cardia carcinoma (GCC) are more common in China, but their causes remain poorly defined.

The occurrence and development of gastrointestinal cancer result from both environmental and genetic factors (2). Previous studies conducted in China have reported that patients with ESCC who had a positive FH of UGI cancer had significantly different gene expression compared with sporadic cases, suggesting that these two types of patients have different genetic backgrounds (5). It has been also reported that factors such as lifestyle and diet habits had an impact on the long-term risk of UGI cancer (6, 7). In etiological research, it is often difficult to distinguish the independent impact of risk factors. Therefore, exploring the combined effects of environmental factors and genetic factors on diseases and analyzing their interaction is very important to cancer prevention in high-risk regions. A family history (FH) of cancer can assist in assessing risk for cancer development, which could represent the effects of both environmental and genetic factors. The American Society of Clinical Oncology (ASCO) recommended that oncologists seeing a new patient should take a family history, specifically asking about cancer in first- and second-degree relatives, to clarify the individual’s risk (8). There is evidence that gastrointestinal cancer such as esophageal cancer, gastric cancer, and colorectal cancer have stronger familial clustering, which could more likely happen in high incidence regions (9–11). Familial esophageal cancer could have a worse prognosis than sporadic esophageal cancer (12). According to a systemic review, some findings of retrospective studies conducted in Europe, East Asia, India, and the United States all verified the association between FH and the risk of gastric cancer mortality (13). However, most of the results were based on case-control studies. Besides, since the environment and lifestyle could be different in high-risk regions, as well as racial differences, conclusions applicable to the general population may not be generalized to high-risk population. Long-time follow-up studies conducting in other high-incidence areas are needed to confirm the reliability of the evidence.

Linxian of Henan Province is one of the high-incidence regions of UGI cancer in China, with a mortality rate exceeding the Chinese average by 10-fold and the American average (in white men) by 100-fold (14). In 1985, Linxian Nutrition Intervention Trial (NIT), the first randomized, double-blind, placebo-controlled nutritional intervention trial, was conducted to explore whether combinations of vitamins and minerals supplements could reduce cancer incidence and mortality. We assumed that FH could increase the risk of UGI cancers (including ESCC, GCC and gastric non-cardia carcinoma (GNCC) in Linxian. Herein, using 30-year follow-up data of the Linxian NIT cohort, we examined the association between a positive history of UGI cancer in a first-degree relative and risk of UGI cancer.

## Materials and Methods

### Study Population

The study design of NIT cohort has been described before (15, 16). In brief, a total of 29,584 adults aged 40 to 69 years were recruited from four northern communes (Yaocun, Rencun, Donggang and Hengshui) in Linxian. Types of nutritional interventions include: A (retinol/zinc), B (riboflavin/niacin), C (vitamin C/molybdenum), and/or D (selenium/vitamin E/beta-carotene), or placebo. After a pre-intervention survey, participants were randomly assigned to one of eight intervention groups, which received daily supplementations including AB, AC, AD, BC, BD, CD, ABCD, or placebo (16). This 2^4^ fractional design can guarantee that half of the subjects received each of the four factors intervention while half of the subjects did not. These daily supplementations lasted for 5.25 years (from March 1986 to May 1991) and doses of the supplement were typically 1.5 to three times the US Recommended Dietary Allowance.

### Family History and Other Exposure Data at Baseline

A baseline questionnaire survey was conducted to collect and assess baseline characteristics and exposure data, including demographic information, lifestyle, dietary habits, FH of cancer, and history of other major diseases. All subjects underwent a brief physical examination to assess their health status. Subjects were asked if their first-degree relatives (parents, siblings, children, and spouses) have been diagnosed with any cancer. Family history of UGI cancer was considered positive if subject had at least one first-degree relative with a history of esophageal or gastric cancer. Smoking was defined as regular cigarettes or hookah or pipe use at least weekly for six months, and alcohol drinking was defined as any alcohol consumed in the previous 12 months. Dietary information included frequency of persimmon bread, moldy bread, foods cooked in oil, meats, eggs, fruit, and vegetable consumption. To avoid the bias caused by seasonal effect, we calculated the frequency of fresh fruit and vegetable consumption in winter/spring and summer/autumn seasons separately.

### Follow-Up of Cancer

During the trial period (1986–1991), village doctors contacted all subjects monthly, and all endpoints were confirmed by an International Endpoints Review Committee (a panel of American and Chinese senior experts). Village investigators continued to contacted participants monthly, and new cancer cases and deaths were confirmed by an international diagnostic team (a panel of American and Chinese diagnosticians, 1991–1996) or senior Chinese experts from Beijing (1996 to 2016). In our study, primary endpoints were ESCC, GNCC, and GCC deaths. GCC was defined as cancer that occurred in the most proximal 3 cm of the stomach and the gastroesophageal junction, and those originated elsewhere in the stomach were defined as GNCC (14).

### Statistical Analysis

Differences in baseline demographic characteristics between FH of UGI cancer categories were compared using Chi-square test for categorical variables and nonparametric test for continuous variables. Participants were censored at the last known follow-up date, lost date of follow-up, date of death, or the closure date for the study (March 2016), whichever came first. Multivariable Cox proportional hazards regression models were used to calculate hazard ratios (HRs) and 95% confidence intervals (95% CI) for the association between FH of UGI and risk of UGI cancer incidence and mortality. Potential covariates in models included: age at baseline (continuous), gender (men or women), smoking status (yes or no), alcohol drinking (yes or no), education level (never, less than 5 years education, primary school, or higher education, and unknown), and frequency of fresh fruit and vegetable consumption (times/year). Subgroup analyses were performed to assess whether the association varied by age at baseline, sex, smoking and alcohol drinking. Sensitivity analyses were also conducted by exclusion of individuals who were followed up less than three years. Cumulative incidence and mortality rates of two groups were estimated by the method of Kaplan–Meier. Log-rank tests were used to examine the significance between cumulative incidence and mortality curves. Statistical analyses were performed using SPSS 23.0. All tests were two sided, and *P <*0.05 was considered statistically significant.

### Ethics Statement

The NIT study was approved by the Institutional Review Boards of the United States (US) National Cancer Institute and Cancer Hospital, Chinese Academy of Medical Sciences (CHCAMS). After being informed of the procedure, general aim, possible benefits, and risks of the study at enrollment, all participants signed the informed consent form as evidence of their willingness to participate in this study. All study procedures followed the Helsinki Declaration.

## Results

After excluding subjects with incomplete baseline data and lost to follow-up, a total of 29,553 participants were included in the final analysis (**Supplementary Figure**). During the 30-year follow-up, a total of 588,401 person-years of follow-up were accumulated, with the median follow up time was 22.56 (0.50–30.08) years. Baseline demographic characteristics, smoking and alcohol drinking status, and frequency of fresh fruit and vegetable consumption for all subjects are shown in **Table 1** according to the FH of UGI cancer categories. Relative to individuals without FH of UGI cancer, participants with a positive FH were mostly men (45.7%), smokers (31.5%), alcohol drinkers (25.6%), and more likely to have higher education level. Most of characteristics varied significantly between the two FH categories (*P <*0.05), except for age at baseline, consumption of fresh fruit and vegetable, and all intervention factors (*P >*0.05).

**Table 1:** Baseline demographic characteristics by family history of UGI cancer in the Linxian General Population NIT Cohort.

	No family history of UGI cancer (N = 20,110)	%	Family history of UGI cancer (N = 9,443)	%	*P* value^*^
Age at baseline, years					0.09
<55	11,951	59.4	5,711	60.5	
≥55	8,159	40.6	3,732	39.5	
Gender					**<0.01**
Women	11,246	55.9	5,132	54.3	
Men	8,864	44.1	4,311	45.7	
Smoking^†^					**<0.01**
No	14,148	70.7	6,465	68.5	
Yes	5,858	29.3	2,978	31.5	
Alcohol Drinking^‡^					**<0.01**
No	15,511	77.5	7,024	74.4	
Yes	4,495	22.5	2,418	25.6	
Education level^‡^					**<0.01**
Never	8,187	40.9	3,625	38.4	
Less than 5 years education	6,223	31.1	2,961	31.4	
Primary school or higher education	3,843	19.2	2,011	21.3	
Unknown	1,753	8.8	846	9.0	
Commune					**<0.01**
Yaocun	6,783	33.7	3,163	33.5	
Rencun	3,716	18.5	2,240	23.7	
Donggang	4,346	21.6	1,914	20.3	
Hengshui	5,265	26.2	2,126	22.5	
Retinol/zinc supplements					0.40
No	10,028	49.9	4,758	50.4	
Yes	10,082	50.1	4,685	49.6	
Riboflavin/niacin supplements					0.39
No	10,008	49.8	4,750	50.3	
Yes	10,102	50.2	4,693	49.7	
Vitamin C/molybdenum supplements					0.88
No	10,060	50.0	4,715	49.9	
Yes	10,050	50.0	4,728	50.1	
Selenium/vitamin E/beta-carotene supplements					0.40
No	10,028	49.9	4,758	50.4	
Yes	10,082	50.1	4,685	49.6	
Fresh vegetable consumption, median (IQR), times/year^†^	730.0 (547.5–912.5)		730.0 (547.5–912.5)		0.34
Fresh fruit consumption, median (IQR), times/year^†^	6.0 (2.0–15.0)		6.0(2.0-13.0)		0.90

^*^P value were derived from χ^2^ or nonparametric tests.

^†^Data on smoking status were not available for 104 subjects (0.4%).

^‡^Data on alcohol-drinking status and education level were not available for 105 subjects (0.4%).

Bold text in this manuscript indicates statistical significance.

There were 5,680 newly diagnosed UGI cancer cases during the follow-up period, with a total of 4,573 UGI cancer deaths occurred, including 2,603 ESCC, 1,410 GCC, and 560 GNCC deaths. **Table 2** presents the associations between FH and risk of UGI cancer incidence and mortality. Participants with FH had a 44 and 27% higher risk of ESCC and GCC incidence (HR_ESCC_ = 1.44, 95%CI: 1.34–1.55; HR_GCC_ = 1.27, 95%CI: 1.15–1.40), as well as a 39 and 26% higher risk of ESCC and GCC mortality compared with those without FH, respectively (HR_ESCC_ = 1.39, 95%CI: 1.28–1.50; HR_GCC_ = 1.26, 95%CI: 1.13–1.40). After adjusting for gender and age at baseline, risk of ESCC mortality in participants with FH was significantly increased by 40% (HR_ESCC_ = 1.40, 95%CI: 1.29–1.51). Higher risk of ESCC and GCC incidence (HR_ESCC_ = 1.45, 95%CI: 1.35–1.56; HR_GCC_ = 1.27, 95%CI: 1.15–1.40) and mortality (HR_ESCC_ = 1.40, 95% CI: 1.30–1.52; HR_GCC_ = 1.27, 95%CI: 1.14–1.42) were also observed after adjusting for age at baseline, gender, smoking status, alcohol drinking, education level, and frequency of fresh fruit and vegetable consumption (times/year). No association was observed for FH of UGI cancer and long-term risk of GNCC incidence and mortality.

**Table 2:** Crude and adjusted hazards ratios (HRs) and 95% confidence intervals (CIs) for the associations between family history of UGI cancer and risk of ESCC, GCC, and GNCC incidence, and mortality.

	UGI Cancer Incidence		UGI Cancer Mortality	
	No family history of UGI cancer	Positive family history of UGI cancer	No family history of UGI cancer	Positive family history of UGI cancer
**ESCC**				
No. of cases/deaths	1,874	1,239	1,583	1,020
Crude HR (95%CI)	1	**1.44 (1.34–1.55)**	1	**1.39 (1.28–1.50)**
Age and gender adjusted HR (95%CI)	1	**1.45 (1.35–1.55)**	1	**1.40 (1.29–1.51)**
Multivariable adjusted HR (95%CI)**^†^**	1	**1.45 (1.35–1.56)**	1	**1.40 (1.30–1.52)**
**GNCC**				
No. of cases/deaths	412	217	371	189
Crude HR (95%CI)	1	**1.14 (0.97–1.35)**	1	1.10 (0.92–1.31)
Age and gender adjusted HR (95%CI)	1	**1.14 (0.96–1.34)**	1	1.10 (0.92–1.31)
Multivariable adjusted HR (95%CI)^†^	1	**1.14 (0.96–1.34)**	1	1.10 (0.92–1.31)
**GCC**				
No. of cases/deaths	1,099	639	890	520
Crude HR (95%CI)	1	**1.27 (1.15–1.40)**	1	**1.26 (1.13–1.40)**
Age and gender adjusted HR (95%CI)	1	**1.26 (1.14–1.39)**	1	**1.26 (1.13–1.40)**
Multivariable adjusted HR (95%CI)^†^	1	**1.27 (1.15–1.40)**	1	**1.27 (1.14–1.42)**

ESCC, esophageal squamous cell carcinoma; GCC, gastric cardia carcinoma; GNCC, gastric non-cardia carcinoma; HR, hazard ratio; 95%CI = 95% confidence interval.

^†^Adjusted for age at baseline, sex, smoking, alcohol drinking, education level, frequency of fresh fruit and vegetable consumption (times/year).

Bold text in this manuscript indicates statistical significance.

**Table 3** presents risk of ESCC, GCC, and GNCC according to family members. Positive FH of both parents and siblings was significantly associated with the increased incidence and mortality of ESCC (Incidence: HR_father_ = 1.36, 95%CI: 1.24–1.49; HR_mother_ = 1.53, 95%CI:1.40–1.68; HR_both parents_ = 1.65, 95%CI 1.40–1.95; HR_brother_ = 1.28, 95%CI:1.09–1.51; HR_sister_ = 1.41, 95%CI: 1.15–1.73; HR_siblings_ = 1.33, 95%CI:1.16–1.52; Mortality: HR_father_ = 1.35, 95%CI: 1.22–1.49; HR_mother_ = 1.48, 95%CI:1.34–1.63; HR_both parents_ = 1.65, 95%CI 1.38–1.97; HR_brother_ = 1.33, 95%CI: 1.12–1.57; HR_sister_ = 1.35, 95%CI: 1.09–1.68; HR_siblings_ = 1.34, 95%CI: 1.16–1.54) and GCC (Incidence: HR_father_ = 1.25, 95%CI: 1.11–1.42; HR_mother_ = 1.23, 95%CI: 1.08–1.40; HR_both parents_ = 1.42, 95%CI: 1.12–1.81; HR_brother_ = 1.31, 95%CI: 1.06–1.63; HR_sister_ = 1.36, 95%CI: 1.04–1.79; HR_siblings_ = 1.33, 95%CI: 1.11–1.59; Mortality: HR_father_ = 1.25, 95%CI: 1.09–1.44; HR_mother_ = 1.23, 95%CI: 1.07–1.42; HR_both parents_ = 1.42, 95%CI 1.09–1.85; HR_brother_ = 1.29, 95%CI: 1.02–1.63; HR_siblings_ = 1.26, 95%CI: 1.03–1.53), respectively. However, participants with FH in sisters did not have significantly higher risk of GCC mortality (HR_sister_ = 1.14, 95%CI: 0.83–1.57). No association of positive FH in spouse between any UGI cancer was observed.

**Table 3:** Risk of ESCC, GCC, and GNCC incidence/mortality according to the types of family members affected^†^.

	UGI Cancer IncidenceHRs (95%CI)			UGI Cancer MortalityHRs (95%CI)		
	ESCC	GNCC	GCC	ESCC	GNCC	GCC
Father	**1.36 (1.24–1.49)**	**1.27 (1.03–1.56)**	**1.25 (1.11–1.42)**	**1.35 (1.22–1.49)**	1.20 (0.96–1.50)	**1.25 (1.09–1.44)**
Mother	**1.53 (1.40–1.68)**	1.02 (0.81–1.28)	**1.23 (1.08–1.40)**	**1.48 (1.34–1.63)**	1.04 (0.82–1.32)	**1.23 (1.07–1.42)**
Both parents	**1.65 (1.40–1.95)**	0.88 (0.53–1.44)	**1.42 (1.12–1.81)**	**1.65 (1.38–1.97)**	0.97 (0.59–1.60)	**1.42 (1.09–1.85)**
Brother	**1.28 (1.09–1.51)**	0.96 (0.64–1.45)	**1.31 (1.06–1.63)**	**1.33 (1.12–1.57)**	0.85 (0.54–1.33)	**1.29 (1.02–1.63)**
Sister	**1.41 (1.15–1.73)**	1.06 (0.64–1.74)	**1.36 (1.04–1.79)**	**1.35 (1.09–1.68)**	1.06 (0.63–1.77)	1.14 (0.83–1.57)
Siblings^‡^	**1.33 (1.16–1.52)**	1.00 (0.72–1.40)	**1.33 (1.11–1.59)**	**1.34 (1.16–1.54)**	0.93 (0.65–1.32)	**1.26 (1.03–1.53)**
Spouse	1.16 (0.96–1.40)	0.68 (0.40–1.16)	1.15 (0.89–1.50)	1.10 (0.89–1.35)	0.76 (0.46–1.28)	1.22 (0.93–1.60)

ESCC, esophageal squamous cell carcinoma; GCC, gastric cardia carcinoma; GNCC, gastric non-cardia carcinoma; HR, hazard ratio; 95%CI, 95% confidence interval.

^†^Adjusted for age at baseline, sex, smoking, alcohol drinking, education level, frequency of fresh fruit and vegetable consumption (times/year).

^‡^Family history of UGI cancer in sibling was defined as participants whose brother or sister has been diagnosed with UGI cancer.

Bold text in this manuscript indicates statistical significance.

Subgroup analysis for the association of FH with risk of ESCC, GNCC, and GCC incidence and mortality are shown in **Tables 4** and **5**. FH of UGI cancer could significantly increase the risk of GCC incidence (HR = 1.31, 95%CI: 1.17–1.47) and mortality in non-drinkers (HR = 1.33, 95%CI: 1.17–1.50). We observed that HRs were varied across strata by sex and smoking (*P_interaction_ <*0.05) for risk of ESCC incidence and mortality, and appeared similar across subgroups by sex, smoking and alcohol drinking for risk of GCC and GNCC incidence and mortality (*P_interaction_ >*0.05).

**Table 4:** Subgroup analyses for family history of UGI cancer and risk of ESCC, GCC and GNCC incidence^†^.

Variables	Number of cases	HRs (95%CI)		*P_interaction_*
**ESCC**				
Age at baseline			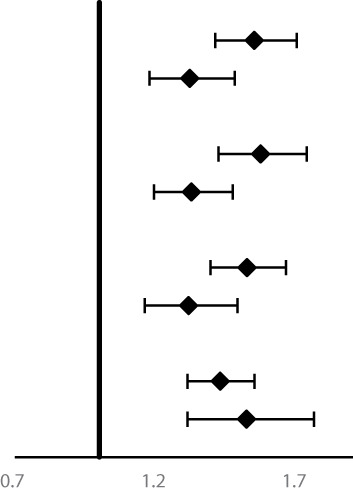	0.05
<55years	1,833	**1.55 (1.41–1.70)**		
≥55years	1,280	**1.32 (1.18–1.48)**		
Gender				**<0.01**
Women	1,625	**1.57 (1.43–1.74)**		
Men	1,488	**1.33 (1.20–1.48)**		
Smoking				**<0.05**
No	2,065	**1.53 (1.40–1.67)**		
Yes	1,038	**1.32 (1.16–1.49)**		
Alcohol drinking				0.44
No	2,378	**1.43 (1.32–1.55)**		
Yes	725	**1.52 (1.32–1.77)**		
**GNCC**				
Age at baseline			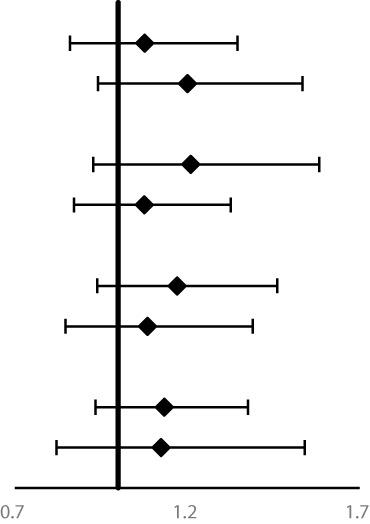	0.47
<55years	344	1.08 (0.86–1.35)		
≥55years	285	1.20 (0.94–1.54)		
Gender				0.39
Women	237	1.21 (0.93–1.58)		
Men	392	1.08 (0.87–1.33)		
Smoking				0.68
No	353	1.17 (0.94–1.46)		
Yes	276	1.09 (0.85–1.40)		
Alcohol drinking				0.92
No	464	1.14 (0.94–1.38)		
Yes	165	1.13 (0.82–1.55)		
**GCC**				
Age at baseline			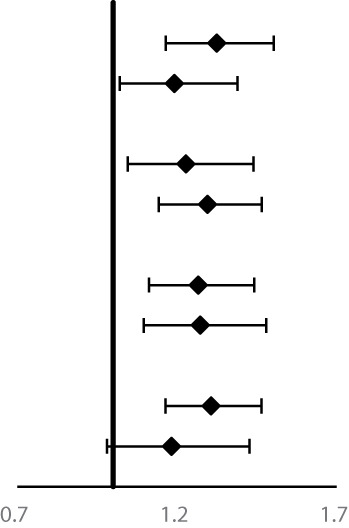	0.36
<55years	1,019	**1.33 (1.17–1.51)**		
≥55years	719	**1.19 (1.02–1.39)**		
Gender				0.91
Women	666	**1.23 (1.05–1.44)**		
Men	1,072	**1.30 (1.15–1.47)**		
Smoking				0.95
No	1,003	**1.27 (1.12–1.44)**		
Yes	728	**1.28 (1.10–1.48)**		
Alcohol drinking				0.46
No	1,267	**1.31 (1.17–1.47)**		
Yes	464	1.19 (0.98–1.43)		

ESCC, esophageal squamous cell carcinoma; GCC, gastric cardia carcinoma; GNCC, gastric non-cardia carcinoma; HR, hazard ratio; 95%CI, 95% confidence interval.

^†^Adjusted for age at baseline, sex, smoking, alcohol drinking, education level, frequency of fresh fruit and vegetable consumption (times/year).

Bold text in this manuscript indicates statistical significance.

**Table 5:** Subgroup analyses for family history of UGI cancer and risk of ESCC, GCC and GNCC mortality^†^.

Variables	Number of deaths	**HRs (95%CI)		*P_interaction_*
**ESCC**				
Age at baseline			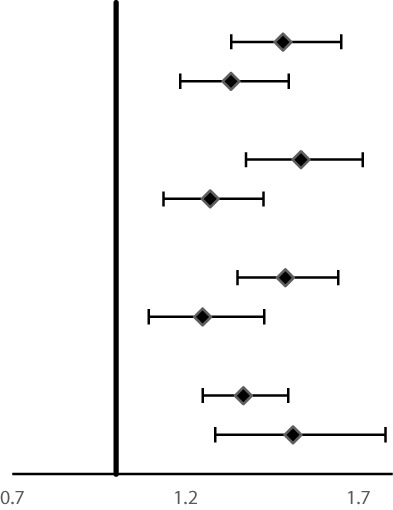	0.24
<55years	1,400	**1.48 (1.33–1.65)**		
≥55years	1,203	**1.33 (1.19–1.50)**		
Gender				**<0.01**
Women	1,317	**1.54 (1.38–1.71)**		
Men	1,286	**1.27 (1.14–1.43)**		
Smoking				**<0.05**
No	1,680	**1.49 (1.35–1.64)**		
Yes	914	**1.25 (1.09–1.43)**		
Alcohol drinking				0.78
No	2,002	**1.37 (1.25–1.50)**		
Yes	592	**1.51 (1.29–1.78)**		
**GNCC**				
Age at baseline			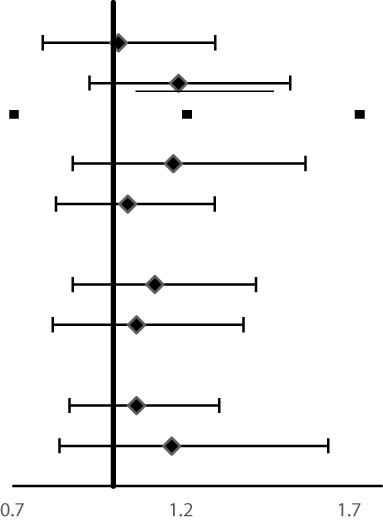	0.34
<55years	281	1.02 (0.79–1.30)		
≥55years	279	1.19 (0.93–1.53)		
Gender				0.38
Women	204	1.18 (0.88–1.57)		
Men	356	1.04 (0.84–1.30)		
Smoking				0.78
No	309	1.12 (0.89–1.42)		
Yes	251	1.07 (0.82–1.39)		
Alcohol drinking				0.71
No	415	1.07 (0.87–1.31)		
Yes	145	1.17 (0.84–1.64)		
**GCC**				
Age at baseline			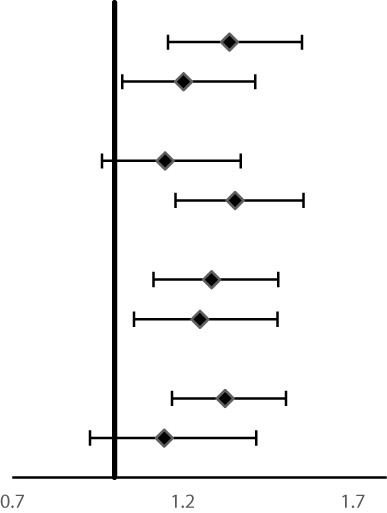	0.44
<55years	762	**1.34 (1.16–1.55)**		
≥55years	648	**1.20 (1.02–1.41)**		
Gender				0.32
Women	552	1.15 (0.96–1.37)		
Men	858	**1.36 (1.18–1.56)**		
Smoking				0.71
No	824	**1.29 (1.12–1.48)**		
Yes	576	**1.25 (1.06–1.48)**		
Alcohol drinking				0.28
No	1,037	**1.33 (1.17–1.50)**		
Yes	366	1.15 (0.93–1.42)		

ESCC, esophageal squamous cell carcinoma; GCC, gastric cardia carcinoma; GNCC, gastric non-cardia carcinoma; HR, hazard ratio; 95%CI, 95% confidence interval.

^†^Adjusted for age at baseline, sex, smoking, alcohol drinking, education level, frequency of fresh fruit and vegetable consumption (times/year).

Bold text in this manuscript indicates statistical significance.

**Figure 1:**
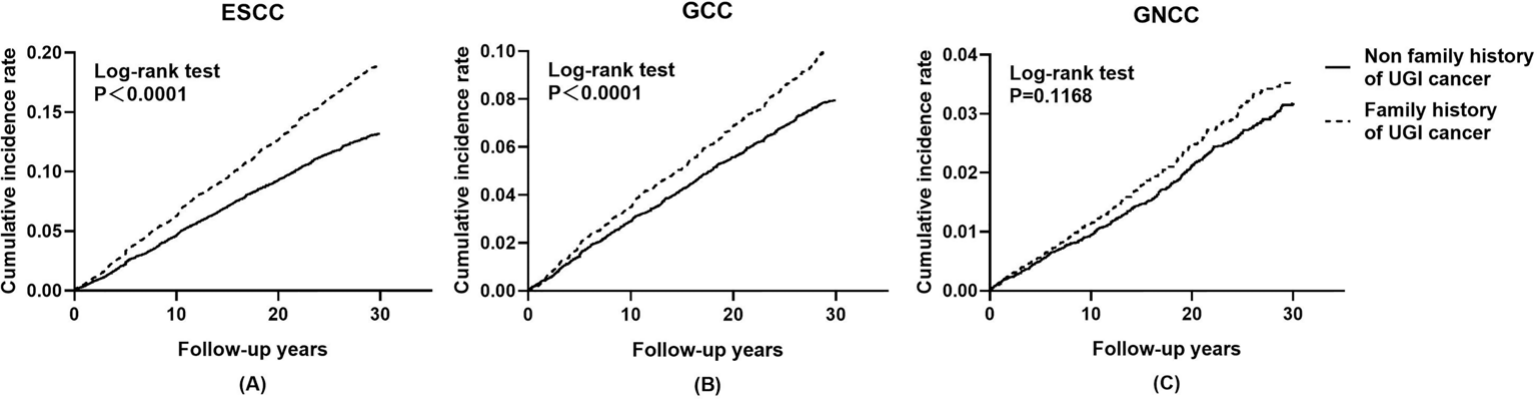
Effect of family history of UGI cancer on cumulative incidence caused by ESCC **(A)**, GCC **(B)**, and GNCC **(C)**. ESCC, esophageal squamous cell carcinoma; GCC, gastric cardia carcinoma; GNCC, gastric non-cardia carcinoma.

**Figure 2:**
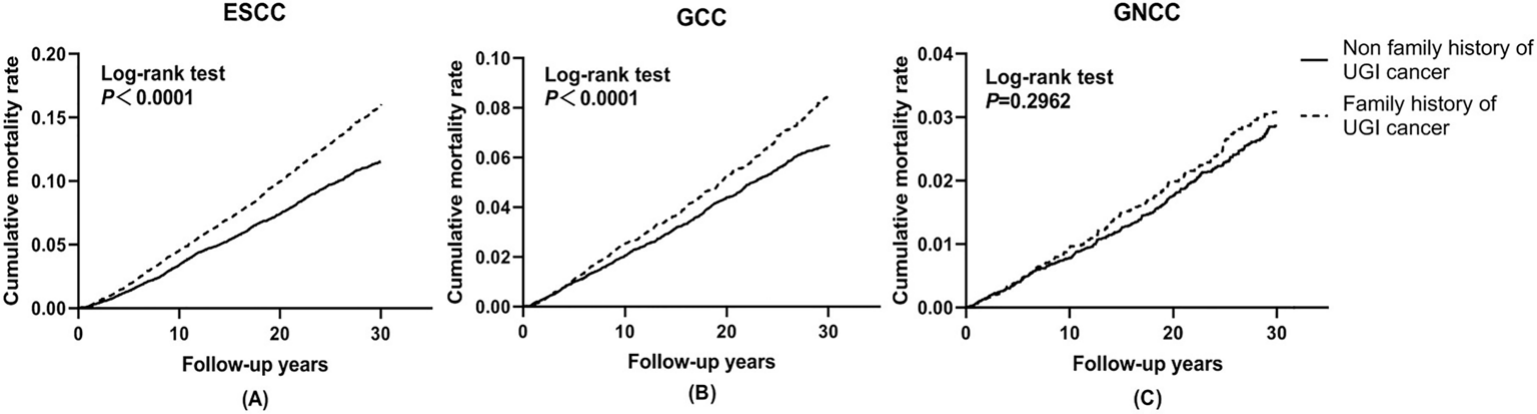
Effect of family history of UGI cancer on cumulative mortality caused by ESCC **(A)**, GCC **(B)**, and GNCC **(C)**. ESCC, esophageal squamous cell carcinoma; GCC, gastric cardia carcinoma; GNCC, gastric non-cardia carcinoma.

Cumulative incidence and mortality curves of ESCC, GCC, and GNCC by FH categories are presented in **Figures 1** and **2**. Compared with the control group, higher cumulative incidence rates (ESCC: 13.0% vs. 18.7%, *P <*0.01; GCC: 7.8% vs. 10.0%, *P <*0.01) and mortality rates (ESCC: 11.5% vs. 16.0%, *P <*0.01; GCC: 6.5% vs. 8.5%, *P <*0.01) of ESCC and GCC were observed in the positive FH group, and no significant difference was observed for GNCC incidence (*P* = 0.12) and mortality (*P* = 0.30).

To avoid reverse causation, sensitivity analysis was used to assess the stability of our results **(** **Table 6** **)**. After exclusion of individuals who were followed up less than three years, HRs for the association between FH of UGI and risk of ESCC, GNCC, and GCC incidence and mortality did not materially change, indicating the robust results of the study analysis.

**Table 6:** Sensitivity analysis by excluding individuals with less than 3 years follow up for the associations between family history of UGI cancer and risk of ESCC, GCC, and GNCC incidence and mortality.

	UGI Cancer Incidence		UGI Cancer Mortality	
	No family history of UGI cancer	Positive family history of UGI cancer	No family history of UGI cancer	Positive family history of UGI cancer
**ESCC**				
No. of cases/deaths	1,534	1,008	1,453	935
Crude HR (95%CI)	1	**1.44 (1.34–1.56)**	1	**1.39 (1.28–1.51)**
Age and gender adjusted HR (95%CI)	1	**1.44 (1.34–1.56)**	1	**1.40 (1.29–1.52)**
Multivariable adjusted HR (95%CI)**^†^**	1	**1.45 (1.35–1.57)**	1	**1.40 (1.29–1.52)**
**GNCC**				
No. of cases/deaths	334	173	325	170
Crude HR (95%CI)	1	1.13 (0.94–1.35)	1	1.12 (0.94–1.36)
Age and gender adjusted HR (95%CI)	1	1.12 (0.94–1.34)	1	1.13 (0.94–1.36)
Multivariable adjusted HR (95%CI)^†^	1	1.12 (0.93–1.34)	1	1.13 (0.94–1.36)
**GCC**				
No. of cases/deaths	877	507	791	468
Crude HR (95%CI)	1	**1.27 (1.14–1.41)**	1	**1.28 (1.14–1.43)**
Age and gender adjusted HR (95%CI)	1	**1.26 (1.13–1.40)**	1	**1.27 (1.14–1.43)**
Multivariable adjusted HR (95%CI)^†^	1	**1.27 (1.14–1.41)**	1	**1.29 (1.15–1.45)**

ESCC, esophageal squamous cell carcinoma; GCC, gastric cardia carcinoma; GNCC, gastric non-cardia carcinoma; HR, hazard ratio; 95%CI, 95% confidence interval.

^†^Adjusted for age at baseline, sex, smoking, alcohol drinking, education level, frequency of fresh fruit and vegetable consumption (times/year).

Bold text in this manuscript indicates statistical significance.

## Discussion

In our large population-based cohort study, we found that a FH of UGI cancer in first-degree relatives was associated with an increased risk of ESCC and GCC incidence and mortality. In the subgroup analyses, FH of UGI cancer could significantly increase the risk of GCC incidence (HR = 1.31, 95%CI: 1.17–1.47) and mortality in non-drinkers (HR = 1.33, 95%CI: 1.17–1.50). No associations were observed for FH of UGI cancer and risk of GNCC incidence and mortality.

Our findings for esophageal cancer were consistent with a similar study conducted in Yangcheng County, Shanxi Province (10), one of the high-risk regions of esophageal cancer in China, suggesting shared susceptibility in esophageal cancer pathogenesis including genetic and environmental factors. However, this study was at odds with reports from Western countries such as US and Sweden (17, 18), where individuals reporting no association between FH of any digestive cancer and risk of esophageal cancer. Possible reasons for this difference may be that squamous cell carcinoma is the most common pathological type of esophageal cancer in China, while adenocarcinoma is common in Western countries, which may lead to differences in genetic susceptibility. Furthermore, genetic factors are more prominent in the development of ESCC in China and family members are more likely to share environmental exposures closely such as diet.

ESCC is a polygenic hereditary disease that can be affected by genetic factors (19, 20). Heritability is defined as the fraction of variation in a phenotype that results from genetic factors in a population (21). Linxian is a high-incidence region of esophageal cancer in China, with an obvious familial aggregation phenomenon. The results of a case-control study showed that the heritability in Linxian (53.4%) was higher than that of non-high-risk areas such as Xi’an (48.14%) and Shanghai (35.7%) (22), indicating that genetic factors may play a greater role in the incidence of esophageal cancer in Linxian than that in non-high incidence areas, and the existence of high incidence areas may be related to genetic factors. The population in Linxian has the following characteristics: 1) family members are concentrated in rural areas; 2) generations have lived in the same village for a long time; 3) low mobility; 4) similar environmental factors; 5) genetic background is clear and stable; 6) lots of family members.

The relationship between FH of cancer and risk of gastric cancer was mostly evaluated using case-control studies. Yaghoobi et al. reviewed case-control studies from 15 countries or regions (13), which consistently showed that gastric cancer history in first-or second-degree relatives was associated with risk of gastric cancer, and the results were not adjusted for risk factors such as age, gender, smoking, drinking, and BMI. There have been several prospective studies undertaken on the role of the FH of malignancy in the development of gastric cancer. A study conducted in Japan showed that a positive FH of cancer, especially stomach cancer, could increase the risk of stomach cancer deaths (23). Minkyo Song et al. indicated that family history of gastric cancer in first-degree relatives was associated with risk of gastric cancer (24), which agreed with our results. However, the positive rate of FH of UGI cancer in the Linxian general population was 32.0%, largely higher than that in other countries such as Iran (5.6%) (25), Spain (6.6%) (26), and the US (4.3%) (4). While most gastric cancers are sporadic, the phenomenon of familial aggregation occurs in about 10% of the gastric cancer cases, of which only 1–3% have a clear hereditary pattern (27), indicating that FH of gastric cancer reflects both environmental factors and genetic susceptibility that are shared within the family. Compared with Western countries, gastric cancer patients and their relatives in China are more likely to be exposed to the common living environment. In Western countries, the living environment does not cause excessive interference with family factors, and the positive rate of family history of gastric cancer may only reflect the role of genetic factors (28, 29).

Our results by anatomic sub-site of gastric cancer (cardia and non-cardia) showed that FH of UGI cancer was associated with increased risk of GCC only. GCC and GNCC are considered as a distinct type of gastric cancers due to their different risk factors for development and prognosis. In a study aiming to clarify the effects of life-style on gastric cancer by subsite focusing on the proximal part (cardia and fundus) and the distal part (pyloric antrum), a declining trend in the risk of gastric cancer was observed from the cardia to antrum (30). One possible explanation may be that the esophagus is anatomically adjacent to the gastric cardia, and it is reasonable to assume that some risk factors affect the cardia more easily than the distal parts (such as middle and antrum), therefore, the esophagus and gastric cardia may share some common etiological factors. Furthermore, GCC and GNCC have significantly different clinicopathological characteristics. A retrospective study showed that GCC patients had larger tumor diameters, higher invasion, and metastasis rates, and the five-year survival rate after a radical resection was significantly lower than that of GNCC patients (31).

In our study, we found higher risk of GCC incidence and mortality in subjects with UGI cancer history in parents compared with those with sisters affected. This finding was contrary to previous studies which suggested that individuals who had a sibling affected were at higher risk of gastric cancer (32, 33). The inconsistency between studies may be explained by the small number of subjects with siblings affected and random variation. Moreover, a positive FH in our study was defined as the subject had at least one first-degree relative with a history of UGI cancer, rather than gastric cancer only. Another possible explanation is that lifestyle and diet habits of parents have a greater impact on children than that of siblings. We found that FH in spouse could not increase the risk of any UGI cancer incidence and mortality. Compared with other types of relatives, subjects and their spouses were not related by blood. Lacking effect of genetic factor may be the potential reason of non-association between FH in spouse and the risk of UGI cancer. Further studies are needed to confirm the association between FH in different type of relatives and gastric cancer risk.

In the subgroup analyses, our results showed the interaction by sex and smoking status for the association between FH and ESCC incidence and mortality, which is contrary to the results of a previous study conducted in Shanxi Province (34). The possible reason may be that some potential confounding factors could not be fully adjusted in subgroup analyses among subjects of different genders or smoking status. For example, we observed the interaction between smoking status and FH of UGI cancer in men and found no interaction between smoking and FH in male subjects (*P_interaction_ >*0.05, data not shown), which may help support our results. We observed the interaction between smoking status and FH of UGI cancer in men and found no interaction between smoking and FH in male subjects (*P_interaction_ >*0.05, data not shown), which means that gender may affect the interaction between smoking status and FH of UGI cancer. Furthermore, differences in sample size and number of cases would also affect our results. Several studies have also explored potential difference in risk among patients with FH of gastric cancer in different genders. Yatsuya and his colleagues revealed that a positive FH of gastric cancer in one or more first-degree relatives might lead to elevated risk of gastric cancer mortality in both genders, whereas a higher risk was observed for women (35). However, studies in Iran (36) and Korea (37) reported higher survival rates of gastrointestinal tract cancer in women, which was consistent with our findings. According to baseline demographic characteristics in the Linxian general population (data not shown), men were more likely to be smokers and alcohol drinkers, indicating that women could have a better lifestyle and be more likely to have a lower risk of GCC, which may lead to increased survival and overestimated adverse effect caused by FH of UGI cancer (7). Until now, it is not verified whether smoking and alcohol drinking modify the association between FH of UGI cancer and gastric cancer mortality, and no effect modification was observed for gastric cancer in our study.

Although environmental factors play an initiating role during tumorigenesis, the occurrence of UGI cancer is caused by both genetic and environmental components. Single nucleotide polymorphisms (SNPs) are one of the most common types of genetic variations in the human genome. From a clinical perspective, SNPs are potential diagnostic and therapeutic biomarkers in UGI cancer (38–40). In recent years, molecular epidemiological studies have found that some enzymes related to the metabolism of carcinogens have genetic and phenotypic polymorphisms in the population, and individuals with different genes or phenotypes have different metabolic capabilities to environmental carcinogens, leading to differences in the probability of individual UGI cancer under the same environmental exposure (41, 42). This may suggest that genetic studies can help persons with an unknown FH of UGI to determine whether they are genetically susceptible to developing UGI cancer.

Our study has several strengths including large number of UGI cancer cases, long-term follow-up, and excellent compliance, which could contribute to more convincing conclusions. However, several limitations need to be noted. Firstly, the major limitation of this study was self-reported data on family history of UGI cancer, which could affect our findings. However, a previous study suggested that the sensitivity and specificity of self-reported family history was fairly good (43). Furthermore, in a prospective cohort study, inaccurate reporting of FH would produce non-differential misclassification that could bias the results towards the null. Secondly, this study was conducted in a nutritionally deprived population, leading to its conclusion may not be extrapolated to a well-nourished population. Thirdly, the study may have selection bias since it is a volunteer study where certain types of people tend to be recruited. Finally, some potential confounders such as Helicobacter pylori infection and socioeconomic status were not included in the analysis.

In summary, in this long-term prospective study, individuals with FH of UGI cancer had higher risk of ESCC and GCC incidence and mortality. For GCC, stronger association was observed for subjects with UGI cancer history in parents than that in siblings. Further studies need to be carried out in order to validate the specific mechanism of FH affecting the risk of UGI cancer.

## Data Availability Statement

The raw data supporting the conclusions of this article will be made available by the authors, without undue reservation.

## Ethics Statement

The studies involving human participants were reviewed and approved by the Institutional Review Boards of Cancer Hospital, Chinese Academy of Medical Sciences. The patients/participants provided their written informed consent to participate in this study. Written informed consent was obtained from the individual(s) for the publication of any potentially identifiable images or data included in this article.

## Author Contributions

HY and J-BW contributed equally to this manuscript. Study concepts: Y-LQ, J-HF, and PT. Study design: Y-LQ, J-HF, and PT. Data acquisition: Y-LQ and J-HF. Quality control of data and algorithms: Y-LQ and J-HF. Data analysis and interpretation: HY and J-BW. Statistical analysis: HY and J-BW. Manuscript preparation: HY and J-BW. Manuscript editing: HY, J-BW, and J-YZ. Manuscript review: HY, J-BW, J-HF, J-YZ, Y-LQ, and PT. All authors contributed to the article and approved the submitted version.

## Funding

This study was supported by a US National Cancer Institute contract (HHSN261201700047C) to the Cancer Hospital, Chinese Academy of Medical Sciences, China.

## Conflict of Interest

The authors declare that the research was conducted in the absence of any commercial or financial relationships that could be construed as a potential conflict of interest.
