# Supplementary material for: Family History and Risk of Upper Gastrointestinal Cancer in the Linxian General Population
Source: Front Oncol. 2021 May 28;11:605106. doi: 10.3389/fonc.2021.605106 (PMC8193945; doi:10.3389/fonc.2021.605106)

Supplementary Material

Supplementary Figure 1. Consort Flow Diagram of the Linxian General Population Trial.

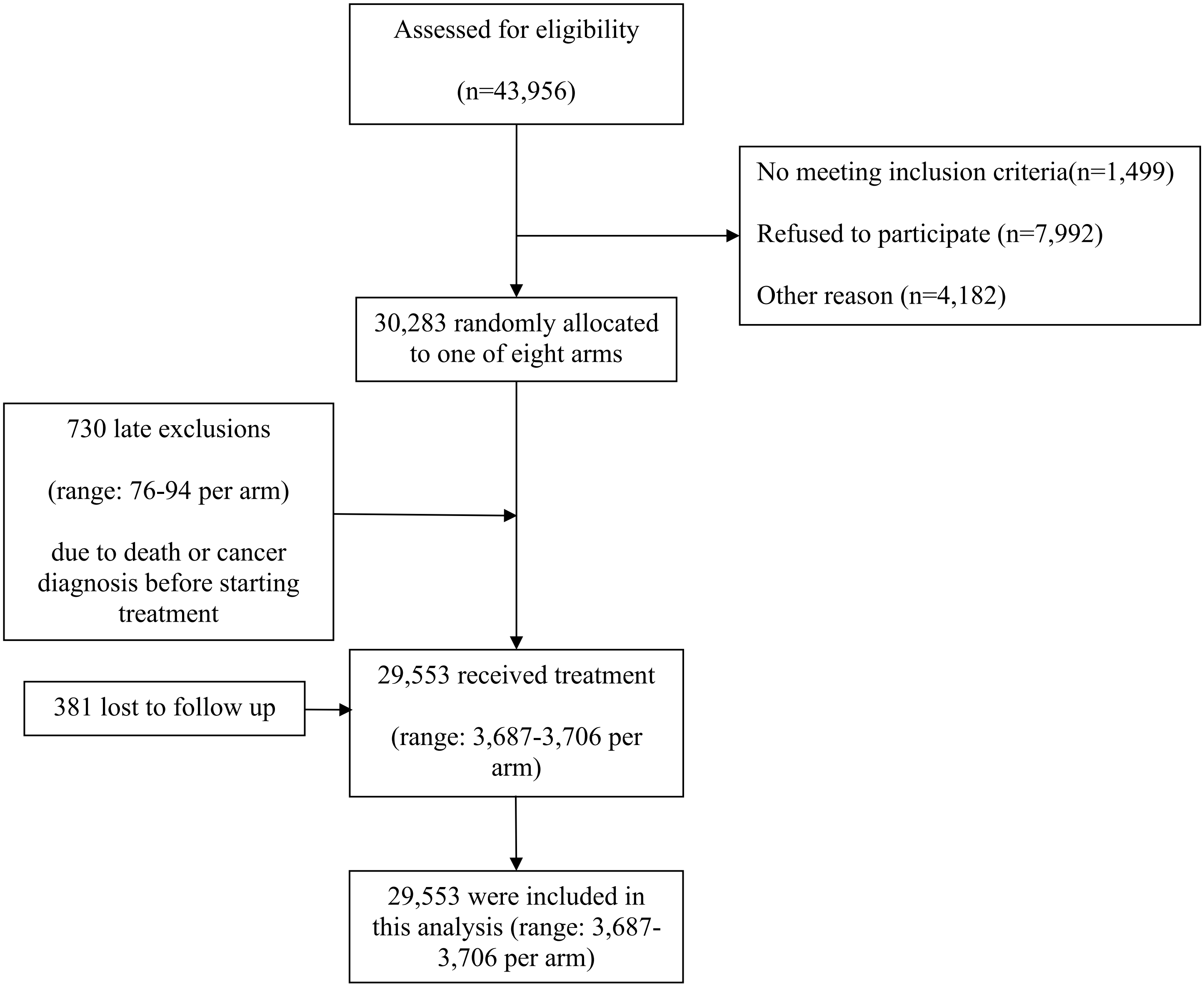

Supplement: Supplementary file 1 [file Image_1.pdf]
